# Fear of COVID-19 lead to procrastination among Turkish university students: The mediating role of intolerance of uncertainty

**DOI:** 10.1186/s40359-021-00681-9

**Published:** 2021-11-10

**Authors:** Hacı Arif Doğanülkü, Ozan Korkmaz, Mark D. Griffiths, Amir H. Pakpour

**Affiliations:** 1 Career Planning, Research and Application Center, Cukurova University, Adana, Turkey; 2 Faculty of Education, Karamanoglu Mehmetbey University, Karaman, 70100 Turkey; 3 International Gaming Research Unit, Psychology Department, Nottingham Trent University, Nottingham, UK; 4 Department of Nursing, School of Health and Welfare, Jönköping University, Barnarpsgatan 39, 55111 Jönköping, Sweden; 5 Social Determinants of Health Research Center, Research Institute for Prevention of Non-Communicable Diseases, Qazvin University of Medical Sciences, Qazvin, Iran

**Keywords:** Fear of COVID-19, Procrastination, Intolerance of uncertainty, University students

## Abstract

**Background:**

The COVID-19 outbreak has not only increased mortality but has also negatively affected mental health among populations across the world. Furthermore, individuals are experiencing uncertainty about their current and future situation because of the pandemic. Therefore, the present study investigated the mediating role of intolerance of uncertainty in the relationship between fear of COVID-19 and procrastination among a sample of Turkish university students.

**Methods:**

Between October and November 2020, 450 university students (291 females and 159 males aged 17 to 24 years) from three state universities in Turkey completed an online survey. Correlation analysis and structural equation modeling methods were employed to examine a model for understanding the general procrastination during COVID-19 pandemic.

**Results:**

The results of the correlation analysis indicated that the fear of COVID-19 was positively correlated with both intolerance of uncertainty (r = .26, p < .001) and procrastination (r = .23, p < .001). The mediation analysis also showed that intolerance of uncertainty had a significant mediating role in the relationship between fear of COVID-19 and procrastination (β = .11, p < .001).

**Conclusion:**

Reducing the fear of COVID-19 and intolerance of uncertainty is likely to contribute to reducing individuals’ procrastination behaviors during the pandemic.

## Introduction

Throughout human history, there have been many pandemics that have been unpredicted and that have had devastating effects on populations [1]. The coronavirus disease-2019 (Covid-19) has quickly become one of the greatest threats and challenges to global public health [2]. COVID-19 is an infectious disease and was first seen in China in December 2019 [3]. As of January 2021, millions of people had been infected with COVID-19 due to its spread to more than 200 countries, regions, or areas, and the virus has killed more than 1.89 million people across the world [4].

To curb the spread of COVID-19, like the rest of the world, the Turkish government implemented various measures during the first few months of 2020. Initially, this included the compulsory wearing of masks, spatial distancing, and various hygiene measures. A limited curfew (permission to go out only at specific times during the day) began to be implemented in all provinces in Turkey in the autumn of 2020. In addition, inter-city travel restrictions were introduced. All social facilities, gyms, and entertainment venues in the country were closed to all activities. During this process, both students and other segments of the population were financially supported by the Turkish government for housing, education and basic needs. All universities in Turkey transformed their education methods from face-to-face education to online education.

Consequently, the COVID-19 pandemic has negatively affected the subjective well-being and led to a fear of COVID-19 among many individuals [5–8]. The fear of COVID-19 has been the cause of many psychological problems, including depression, anxiety and stress among individuals [9, 10]. These psychological problems are known to be important predictors of procrastination behaviors that negatively affect individuals’ effective use of time [11] and their state of happiness [12]. Therefore, the fear of COVID-19 appears to be an important factor that affects procrastination behavior among individuals.

Procrastination is viewed as a voluntary delay of an action and can bring about negative consequences [13, 14]. Procrastination, which occurs among approximately half of university students at some point during their studies [14], is associated with mental health indices, including life satisfaction [15], emotional well-being [16, 17], self-efficacy and self-regulation [18], resilience [19], and self-esteem [20], as well as depression, anxiety, and stress [21]. It can also lead to negative effects on individuals' professional and academic performance [14]. Moreover, procrastination has also been associated with it delaying people’s use of health services [22]. Individuals with procrastination behavior face difficulties in issues that cover most life aspects, such as emotion regulation [23], time management [11], and learning strategies [24].

One of the consequences that COVID-19 has had on individuals is uncertainty [25]. It is not known when life will return to normal after COVID-19 pandemic, and this can cause uncertainty among individuals [26]. Individuals’ intolerance of uncertainty increases with increased fear and anxiety [26]. In this case, individuals want to understand threatening situations and to have a sense of control over these situations [27]. However, the continuing uncertainty regarding COVID-19 can enhance fear reactions and the intolerance of uncertainty among some individuals [28].

Intolerance of uncertainty is defined as the tendency to respond negatively to uncertain events and situations in cognitive, emotional, and behavioral terms [29]. This response arises irrespective of whether the uncertainty is positive or negative [30]. Intolerance of uncertainty is also associated with individuals’ mental health and is one of the important predictors of psychological well-being [27]. Psychological problems, such as depression, anxiety, and stress [21, 31] are all associated with intolerance of uncertainty [32, 33]. Individuals with high intolerance of uncertainty can be said to experience emotional distress [34]. Moreover, intolerance of uncertainty is associated with various behaviours in the life of the individual, such as social skills [35], academic performance [36], professional burnout [37], and happiness [38].

### The present study

Previous research has shown that fear of COVID-19 increases individuals' intolerance of uncertainty [26, 27]. In addition, the fear of COVID-19 is positively associated with depression, anxiety, and stress, which are important predictors of individuals' procrastination behaviors [10, 26]. Moreover, intolerance of uncertainty is predictive of procrastination [39]. Considering the extant literature, it was hypothesized that intolerance of uncertainty may act as a mediator between fear of COVID-19 and procrastination. Therefore, the present study examined this specific relationship.

**Fig. 1:**
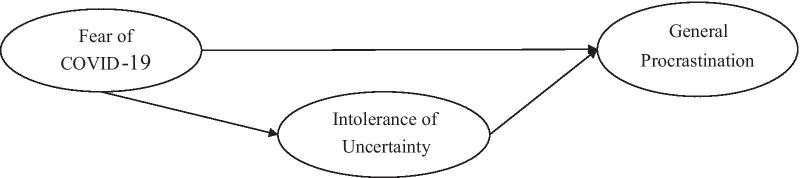
The hypothesized structural model

The present study was carried out with Turkish university students. One of the reasons for specifically conducting the study with Turkish university students is that 14% of COVID-19 patients are young adults in Turkey. Additionally, it is observed that the morbidity rate among young adults is equal for males and females. The mortality rate of patients in Turkey is 2.67%, and among young adults, this rate is 0.04% [40]. Moreover, the COVID-19 pandemic has negatively affected university students in many ways including both physical and mental health [41, 42]. Consequently, the present study assessed whether procrastination is affected by the fear of COVID-19 among Turkish university students. It also investigated the mediating role of intolerance of uncertainty in the relationship between the fear of COVID-19 and general procrastination behavior. The hypothetical model of the present study is presented in Fig. 1. In the present study, the following hypotheses (H_s_) were examined:

#### H_1_

Fear of COVID-19 will positively predict the general procrastination.

#### H_2_

Fear of COVID-19 will positively predict the intolerance of uncertainty.

#### H_3_

Intolerance of uncertainty will positively predict general procrastination.

#### H_4_

Intolerance of uncertainty will have a mediating role in the relationship between fear of COVID-19 and general procrastination.

## Methods

### Participants, procedure, and ethics

The study’s participants comprised 450 individuals (291 females [64.7%], 159 males [35.3%]), with ages ranging from 17 to 24 years (mean age = 19.63 years, SD = 1.57). A total of 53 participants had a low socio-economic status (11.8%), 339 participants were medium status (75.3%), and 58 participants were high status (12.9%). Just over one-quarter of the participants (26.9%; n = 121) had been diagnosed with COVID-19 at some point prior to or at the time of data collection.

Using convenience sampling method, the data were collected online via *Google Forms* from state universities in the cities of Karaman, Adana, and Nigde between October and November 2020. Data collection took approximately 15 minutes per participant. While collecting the data, no personally identifiable information was requested from the participants. The study was performed in accordance with the Declaration of Helsinki and was approved and registered by the ethical and research committee from Ethics Committee of Karamanoglu Mehmetbey University (E-95728670-044-E.26034). All participants provided written informed consent.

### Measures

#### Fear of COVID-19 Scale (FCV-19S)

The seven-item FCV-19S [5, 26] assesses the fear of COVID-19. The scale is uni-dimensional and its items (e.g., “I am most afraid of coronavirus-19”) are responded to on a five-point Likert type scale from 1 (*strongly disagree*) to 5 (*strongly agree*)*.* The higher the score the greater the fear of COVID-19. The internal consistency of the scale, as assessed by Cronbach alpha, was found to be very good in the present study (α = 0.86).

#### Intolerance of Uncertainty Scale (IUS-12)

The 12-item scale IUS-12 [43, 44] assesses the tendency of individuals to react emotionally, cognitively, and behaviorally to uncertain events and situations. The scale comprises two dimensions with six items in each subscale (e.g., “Unforeseen events upset me greatly” [prospective anxiety]), “Uncertainty keeps me from living a full life” [inhibitory anxiety]). Items are responded to on a five-point Likert type scale from 1 (*Not at all characteristic of me*) to 5 (*Entirely characteristic of me*). A higher score indicates greater intolerance of uncertainty. The internal consistency of the scale, as assessed by Cronbach alpha, was found to be very good in the present study (α = 0.87).

#### General Procrastination Scale (GPS)

The 18-item GPS [45] assesses the extent to which individuals do their daily work or not. The scale comprises two dimensions with nine items in each subscale (e.g., “My family and friends always say that I do things at the last minute” [procrastination], “I finish things on time” [using time effectively]). Items are responded to on a five-point Likert type scale from 1 = (*Doesn't reflect me at all*) to 5 (*Reflects me completely*). A higher score indicates a greater general tendency to procrastinate. The internal consistency of the scale, as assessed by Cronbach alpha, was found to be excellent in the present study (α = 0.95).

### Data analysis

Descriptive statistical analyses were used to investigate socio-demographic data. Independent samples *t*-tests and ANOVAs (one-way analyses of variance) were used to examine the descriptive findings. Pearson’s correlation analysis was used to investigate the relationship between fear of COVID-19, intolerance of uncertainty, and general procrastination. A structural equation model using full information maximum likelihood estimation was conducted to assess the mediating role of intolerance of uncertainty in relationship between fear of COVID-19 and procrastination. As Fig. 1 shows, in the hypothetical model, fear of COVID-19 was considered to be an endogenous latent variable while intolerance of uncertainty was considered as the latent variable acting as a mediator, and general procrastination was the latent and dependent variable. Model fit indices were evaluated using the following indices: χ^2^/df, Comparative Fit Index (CFI), Tucker-Lewis Index (TLI), Root Mean Square Error of Approximation (RMSEA) and Standardized Root Mean Squared Residual (SRMR). χ^2^/df < 5, TLI > 0.90, CFI > 0.90, and RMSEA and SRMR < 0.08were used to determine whether the model fit the data [46–48]. Bootstrapping tests were also performed to examine whether intolerance of uncertainty mediated the relationship between the fear of COVID-19 and general procrastination [49]. A total of 10,000 resampling and 95% confidence intervals were used in this process. IBM SPSS Statistics 25 and AMOS Graphics 24 software packages were used for the analysis of the data.

## Results

### Descriptive statistics

Findings concerning the descriptive variables are presented Table 1. There were no significant differences in fear of COVID-19 scores on any of the descriptive variables (i.e., “having a COVID-19 diagnosis”, socio-economic status, and gender).

**Table 1:** Descriptive statistics among participants (n = 450)

	*n*	%	*M*	*SD*
*Gender*^*1*^				
Female	291	64.7	19.42	5.46
Male	159	35.3	18.35	5.80
*Socio-economic status*^*2*^				
Low	53	11.8	19.21	6.66
Medium	339	75.3	19.22	5.58
High	58	12.9	17.81	4.48
*Having a COVID-19 diagnosis*^*3*^				
Yes	121	26.9	18.9	5.70
No	329	73.1	19.1	5.57

1: t(448) = 1.939, p = .053; 2: F (2, 447) = 1.618, p = .199; 3: t(448) = − .154, p = .878

### The relationships between fear of COVID-19, intolerance of uncertainty, and general procrastination

Pearson’s correlation analysis showed that the fear of COVID-19 was positively correlated with the intolerance of uncertainty (*r* = 0.26, *p* < 0.001), and general procrastination (*r* = 0.23, *p* < 0.001), whereas intolerance of uncertainty was positively correlated with general procrastination (*r* = 0.24, *p* < 0.001).

### Findings of the hypothesized mediation model

**Fig. 2:**
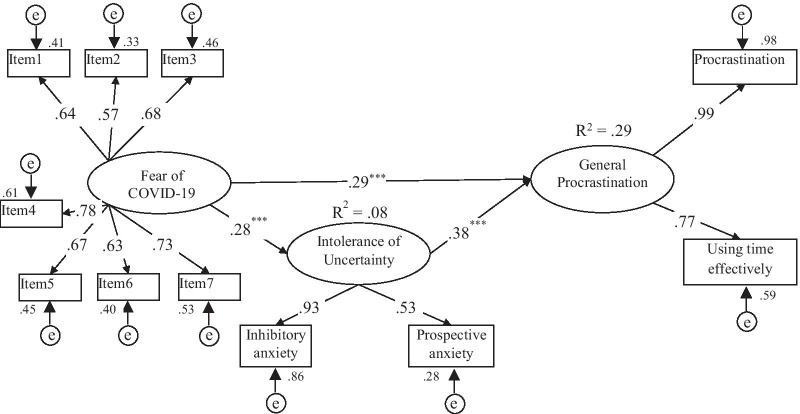
Mediation model of the relationships between the research variables

The results of the hypothesized mediation model are presented in Fig. 2. The goodness of fit indices of the model were found to be significant [χ^2^ (39, N = 450) = 135.99; *p* < 0.001; χ^2^/df = 3.49; TLI = 0.92; CFI = 0.92; RMSEA = 0.08 (LO = 0.06, HI = 0.09]. The path coefficients of fear of COVID-19 and intolerance of uncertainty (β = 0.28, *p* < 0.001), fear of COVID-19 and general procrastination (β = 0.29, *p* < 0.001), intolerance of uncertainty and general procrastination (β = 0.38, *p* < 0.001) were statistically significant. The indirect effect of the fear of COVID-19 in predicting general procrastination via the mediation of intolerance of uncertainty in the model was also found to be significant (β = 0.11, *p* < 0.001). The fear of COVID-19 and intolerance of uncertainty explained 29% of the variance of general procrastination (R^2^ = 0.29, *p* < 0.001).

While performing bootstrapping analysis, 95% confidence interval and 10,000 resampling paths were conducted. The results are presented Table 2. All the path coefficients in the model were statistically significant (Table 2). On the other hand, the range of lower and upper limit values were not zero. Therefore, intolerance of uncertainty had a mediating role between fear of COVID-19 and general procrastination.

**Table 2:** Bootstrapping test of the mediating effect of fear of COVID-19 on general procrastination

Pathways	Coefficient	Standard error	95% CI
*Direct effect*			
Fear of COVID-19 → Intolerance of uncertainty	.28	.07	.17 to .40
Fear of COVID-19 → General procrastination	.29	.06	.19 to .39
Intolerance of uncertainty → General procrastination	.38	.06	.29 to .47
*Indirect effect*			
Fear of COVID-19 → Intolerance of uncertainty → General procrastination	.11	.03	.06 to .17
*Total effect*			
Fear of COVID-19 → General procrastination	.40	.06	.30 to .50

*CI*: confidence interval. All path coefficients were significant at *p* < .001

## Discussion

The present study is noteworthy because it is the first one to examine the effect of the fear of COVID-19 on procrastination. It is also important in that it examined the effect of intolerance of uncertainty on the relationship between procrastination and COVID-19. It is thought that while alleviating the fear of COVID-19 among university students is of importance, reducing their intolerance of uncertainty can also reduce their procrastination behaviors during the COVID-19 pandemic.

The study assessed the extent to which university students' fear of COVID-19 and their intolerance of uncertainty predicted their procrastination behavior using structural equation modeling. According to the results of the study, fear of COVID-19 and intolerance of uncertainty were found to significantly predict procrastination. Moreover, university students' intolerance of uncertainty had a mediating role in the relationship between the fear of COVID-19 and procrastination.

One of the key findings of the present study was that the fear of COVID-19 positively predicted procrastination. Previous studies have shown that the fear of COVID-19 can cause psychological problems, such as depression, anxiety, and stress [9, 10, 36, 50]. The extant literature has indicated that psychological problems have a strong effect upon procrastination [21, 31, 51]. Therefore, it can be said that individuals appear to procrastinate due to psychological problems that the COVID-19 pandemic has created. Individuals’ procrastination of their activities, especially in public areas (shopping, bill payment and banking etc.), may be due to the threat of infection because COVID-19 has disrupted individuals’ normal lifestyles, and the risk of infection has made it difficult for individuals to leave their immediate social environments [52].

Another finding was that the fear of COVID-19 positively predicted intolerance of uncertainty. No vaccine or drugs had been developed against COVID-19 at the time of data collection, and the accumulative death toll due to the disease was increasing daily. Experts cannot predict when the fight against COVID-19 will be successful and when life will return to its normal course, which causes uncertainty [26]. Uncertainty increases fear, and fear increases the individual's intolerance of uncertainty [26, 27]. Therefore, it can be said that the fear of COVID-19 is a facilitating factor that increases individuals’ intolerance of uncertainty.

Another finding of the study was that intolerance of uncertainty positively predicted procrastination. It is known that intolerance of uncertainty is also a facilitating factor in procrastination [39]. One of the reasons for procrastination is the avoidance behaviors of the individual [53]. The motivation of the individual for avoidance increases due to various factors, such as fear and anxiety, and this can lead to procrastination. On the other hand, among individuals with a high level of intolerance of uncertainty, the avoidance response functions as a coping strategy, and individuals use the avoidance response in the face of uncertainties that make them feel psychologically uncomfortable [54]. Therefore, the finding here that intolerance of uncertainty positively predicts procrastination can be explained by the avoidance responses of the individuals.

Moreover, the findings of the present study indicated that intolerance of uncertainty played a mediating role in the relationship between the fear of COVID-19 and procrastination. This finding suggests that as university students' fear of COVID-19 decreases, their intolerance of uncertainty decreases, and that they will engage in fewer procrastination behaviors. Based on the findings here and the extant literature, it can be said that psychological problems due to the COVID-19 pandemic will increase procrastination [21, 31, 51]. However, in addition to the relationship between COVID-19 and procrastination, the uncertainties concerning COVID-19 and the unpredictability of what will happen in the future [9, 39] indicate that intolerance of uncertainty plays a mediating role in increased procrastination behaviors due to COVID-19.

The results of the present study have important implications. The study contributes to the COVID-19 literature, where there are many more unknowns, by demonstrating the effect of COVID-19 on procrastination. Procrastination is a process related to the well-being of individuals [16, 17]. Therefore, the study here is important in that it demonstrates the impact of COVID-19 on procrastination, a factor associated with consequence to psychological well-being. On the other hand, it is known that many epidemic diseases have been experienced worldwide throughout human history [1, 55]. As previous research demonstrates, pandemic conditions often cause negative emotions [56, 57]. However, it can be said that all kinds of fearful situations (poor health, other pandemics, loss of employment, economic downturns, etc.) that increase uncertainty such as COVID-19 will also have a negative effect on the mental health of individuals. Therefore, the findings of the present study may be valid not only during the COVID-19 pandemic period, but also in situations where fear affects individuals.

With online intervention programs, studies can be conducted to reduce the psychological problems of individuals caused by COVID-19. Satisfying social needs with video chats with family members or friends appears to be effective in reducing the feeling of fear [58, 59]. Therefore, individuals can be encouraged to have online interactions. Counseling lines can be established so that individuals with a high level of intolerance of uncertainty can obtain accurate information concerning COVID-19 at any time. Consequently, uncertainty regarding the pandemic can be clarified. Moreover, intervention programs that can reduce procrastination behaviors due to the fear of COVID-19 can be developed. Recommended intervention studies can be applied to students through university psychological counseling and guidance centers.

The present study has some limitations. It was carried out with students enrolled in three different state universities in Turkey. The generalizability of the results could therefore be increased by carrying out similar studies with university students who have different cultural characteristics from different cultures and countries. The lack of information about the perceived social support of the participants in this study is also a limitation because perceived social support is a factor associated with fear of COVID-19 [60] and intolerance of uncertainty [61]. No information was collected from participants about whether they had received psychological support to cope with the fear of COVID-19. This is also a limitation because it is a factor that may have impacted study findings. In order to examine the effect of fear of COVID-19 on individuals' procrastination behaviors, new research models can be designed as mediators and modifiers such as hope and optimism which are positive concepts for the future. In addition, new research designs based on qualitative paradigms could be implemented. In these studies, the cognitive and emotional effects of fear of COVID-19 on individuals' procrastination behaviors could be examined in depth.

The present study was conducted with university students and was carried utilizing a cross-sectional design. Similar studies could be conducted among different employee groups to investigate whether the fear of COVID-19 and intolerance of uncertainty have an effect on procrastination behaviors of working individuals rather than academic work. In addition, the present study showed that COVID-19 fear appears to explains its effect on procrastination via intolerance of uncertainty. In future studies, a similar model could be created to include different fears or fear situations. By comparing the results to those in the present study, the accumulative findings will significantly add to the literature on the psychological effects of the epidemic.

## Conclusion

The present study found that the fear of COVID-19 affected individuals' procrastination behaviors. Also, intolerance of uncertainty was determined to be an important facilitating factor in procrastination behaviors among university students. According to the results of the study here, intolerance of uncertainty plays an important mediating role in the relationship between the fear of COVID-19 and procrastination. Findings indicated that during the pandemic period, when uncertainties and fears are common, procrastination behaviors should be taken into account when developing interventions to prevent negative effects among individuals.

## Data Availability

The datasets used and/or analyzed during the current study are available from the corresponding author on reasonable request.
